# Interlaboratory Study on Zebrafish in Toxicology: Systematic Evaluation of the Application of Zebrafish in Toxicology’s (SEAZIT’s) Evaluation of Developmental Toxicity

**DOI:** 10.3390/toxics12010093

**Published:** 2024-01-22

**Authors:** Jon T. Hamm, Jui-Hua Hsieh, Georgia K. Roberts, Bradley Collins, Jenni Gorospe, Barney Sparrow, Nigel J. Walker, Lisa Truong, Robyn L. Tanguay, Sylvia Dyballa, Rafael Miñana, Valentina Schiavone, Javier Terriente, Andrea Weiner, Arantza Muriana, Celia Quevedo, Kristen R. Ryan

**Affiliations:** 1 Inotiv, P.O. Box 13501, Research Triangle Park, NC 27709, USA; 2 Division of Translational Toxicology, National Institute of Environmental Health Sciences, Research Triangle Park, NC 27709, USA; 3 Battelle Memorial Institute, Columbus, OH 43201, USA; 4 Department of Environmental and Molecular Toxicology, The Sinnhuber Aquatic Research Laboratory, The Environmental Health Sciences Center, Oregon State University, Corvallis, OR 97331, USA; 5 ZeClinics SL., 08980 Barcelona, Spain; 6 CTI Laboratory Services Spain SL., 48160 Bilbao, Spain; 7 BBD BioPhenix SL. (Biobide), 20009 San Sebastian, Spain

**Keywords:** zebrafish, optimization, harmonization, high-throughput screening, developmental toxicity, interlaboratory study

## Abstract

Embryonic zebrafish represent a useful test system to screen substances for their ability to perturb development. The exposure scenarios, endpoints captured, and data analysis vary among the laboratories who conduct screening. A lack of harmonization impedes the comparison of the substance potency and toxicity outcomes across laboratories and may hinder the broader adoption of this model for regulatory use. The Systematic Evaluation of the Application of Zebrafish in Toxicology (SEAZIT) initiative was developed to investigate the sources of variability in toxicity testing. This initiative involved an interlaboratory study to determine whether experimental parameters altered the developmental toxicity of a set of 42 substances (3 tested in duplicate) in three diverse laboratories. An initial dose-range-finding study using in-house protocols was followed by a definitive study using four experimental conditions: chorion-on and chorion-off using both static and static renewal exposures. We observed reasonable agreement across the three laboratories as 33 of 42 test substances (78.6%) had the same activity call. However, the differences in potency seen using variable in-house protocols emphasizes the importance of harmonization of the exposure variables under evaluation in the second phase of this study. The outcome of the Def will facilitate future practical discussions on harmonization within the zebrafish research community.

## 1. Introduction

Zebrafish (*Danio rerio*), a small tropical fish native to the southeastern Himalayan region, can be easily maintained and bred in a laboratory setting [1,2,3]. Zebrafish have high fertility rates, rapid development, a short intergenerational time, and a completely annotated genome that is highly concordant with mammalian species [4,5]. For these reasons, zebrafish have been used extensively as a model organism in several scientific fields, including general toxicology [6,7,8]; developmental toxicology [9,10]; behavioral toxicology [11]; drug discovery [12,13,14]; and ecotoxicology [15,16,17].

To replace, reduce, or refine animal use [18,19], the zebrafish embryo model has been investigated as a humane replacement for adult fish [20] and adopted for acute toxicity testing [21]. Brannen et al. [22] developed the zebrafish embryo teratogenicity test (ZET), which included a morphological scoring system for the characterization of teratogenicity. Since then, the use of zebrafish embryos has expanded rapidly, with many laboratories developing in-house scoring systems [6,7,10,23,24,25,26].

The use of zebrafish embryos has several advantages in toxicology studies, including the ability of a breeding colony to generate thousands of developmentally synchronized embryos per day [27,28]. Embryos with an intact chorion are approximately 1 to 1.5 mm in diameter [29,30], making them easy to maintain and treat in the 96-well plates that are commonly used in medium- to high-throughput platforms [31]. The chorion is transparent, allowing for direct microscopic observation and evaluation throughout the entire developmental process, the stages and timing of which are well documented [28,30,32]. Numerous laboratories and groups are working to develop [33,34,35] and harmonize [9,23] zebrafish embryo screening models as an alternative to traditional in vivo developmental toxicity screening [36,37]. New alternative models should be reproducible and transferable, compatible test substances and limits of detection and quantification should be defined, and the method should provide accurate results [38]. There are considerations for using any test system, including zebrafish, as a model for human health, including differences in the pharmacological effects of drugs [12] and the fact that the phylogenetic distance from humans results in anatomy and physiology differences [13]. However, zebrafish offer the opportunity to rapidly screen chemicals in an intact vertebrate, which, despite some differences, has numerous similarities in anatomy and physiology to humans and a sequenced genome in which 70% of human genes have at least one zebrafish ortholog [4].

In 2014, a Collaborative Workshop on Aquatic Models and 21st Century Toxicology [39] was organized by multiple organizations to discuss how aquatic models may be used to screen and prioritize substances for further in vivo testing, how the mechanisms of toxicity are assessed, and how the data gathered can impact environmental and human health. Significant discussions focused on the lack of standardization of the exposure protocols, data capture methods, and scoring systems for aquatic models and how inconsistencies can create variable data outputs and impede data utilization, and, in some cases, the acceptance of the model. The workshop participants agreed that the development of standardized protocols, validation, and subsequent regulatory acceptance would facilitate greater usage of aquatic models in toxicology. In response to these discussions, scientists in the Division of Translational Toxicology (DTT) (formerly the Division of the National Toxicology Program (DNTP)) at the National Institute of Environmental Health Sciences (NIEHS) developed the Systematic Evaluation of the Application of Zebrafish in Toxicology (SEAZIT) program (the SEAZIT website is accessible at: https://ntp.niehs.nih.gov/go/seazit accessed on 15 January 2024) to investigate sources of variability using the zebrafish embryo model and provide a scientific foundation for making programmatic decisions on the further use of zebrafish in the toxicological screening and prioritization of test substances for more targeted evaluations.

One of SEAZIT’s goals is understanding the sources of variability in biological effects. In the first SEAZIT project, several distinguished researchers using zebrafish embryos for toxicology testing were asked to participate in an information-gathering group. Group members were provided a questionnaire to collect information on their testing protocols, including zebrafish strains, types of feed, preparation of system water, disease surveillance practices, embryo exposure conditions, and the endpoints assessed. Discussions with the information-gathering group identified the study design parameters that could potentially influence the study outcomes for test substance screening using zebrafish embryos, with the results published in Hamm et al., 2019. Those key design parameters identified by the group that could affect outcomes were (1) whether the chorion is left intact or removed before exposure to test substances, and (2) whether the exposure media are static or renewed every 24 h (static versus static renewal exposure). The chorion is known to be a semipermeable membrane and its influence on the test substance uptake has not been completely characterized [40,41]. Renewal of the exposure media was thought to be a key parameter, as fresh media would provide additional test substance that could accumulate in the embryo or replace the old solution, in which the test substance had degraded.

To test the influence of the chorion and renewal of the exposure media on test substance activity and potency, an interlaboratory study was designed. Three unique laboratories were selected to test 42 substances in two phases: an initial dose-range-finding study, (DRF) followed by a definitive study (Def). This manuscript is meant to serve two purposes. First, we aim to briefly describe the overall design and rationale for the interlaboratory study. Second, we highlight the outcome of the DRF phase of the study. In the DRF study, the three laboratories utilized their in-house protocols to refine the dose ranges for the Def study. This approach allowed us to assess the laboratories’ capacity to conduct the screening using their established protocols before progressing to the more intricate Def phase. This study also provided valuable data to assess the lab-specific testing performance (i.e., intralaboratory reproducibility) and chemical potency. This design also allowed us to mimic the variability which is likely observed across laboratories (i.e., interlaboratory) with different protocols. The lessons learned from the DRF study emphasize the potential benefits of standardized testing protocols for the zebrafish research community interested in toxicology testing.

## 2. Materials and Methods

### 2.1. Test Substance Selection

A screening library was selected from the test substances present in the ToxCast^TM^ library (available at https://www.epa.gov/chemical-research/exploring-toxcast-data accessed on 15 January 2024), which represent a variety of physicochemical and biological activity. In terms of physicochemical properties, we collected logP and water solubility as well as molecular weights from ChemSpider and PubChem (available at http://www.chemspider.com/ and https://pubchem.ncbi.nlm.nih.gov/, accessed on 15 January 2024, respectively). Padilla [6] reported that logP correlates with the likelihood that a substance is toxic in zebrafish, as well as its potency. Within the ToxCast^TM^ library of test substances, we also examined volatile substances so that we could assess the impact of volatility on toxicity in this test system. A range of sources was used to compile information on the biological activity of the substances in our list, with particular attention paid to chemicals affecting pathways important to embryonic development, including vascular development and the endocrine system [42]. A small number of substances were selected based upon recommendation by the information group. In the experience of that group, the toxicity of these substances is influenced by the exposure scenario utilized. The positive control from the Fish Embryo Acute Toxicity (FET) test, 3,4-dicholoroaniline, was selected as the positive control in the current study based on its extensive use as a positive control in the FET [21].

Using the data generated in ToxCast^TM^, we highlighted substances (see Supplemental Table S1) that were vascular disruptors [43,44,45], androgen receptor agonists and antagonists, active in-cell stress or cytotoxicity assays [46], and estrogen receptor agonists and antagonists [47], as well as substances that were previously tested in zebrafish [6], in order to provide a broad array of biological activities. In addition to the testing in zebrafish embryos as part of ToxCast^TM^, several substances were previously tested in zebrafish embryos by DTT [48,49,50]. To provide in vivo reference data, we collected rat and rabbit developmental reproductive summary scores from the U.S. EPA’s Toxicity Reference Database [51] and recorded which substances had developmental toxicity studies in rodents from DTT or OECD. We used the developmental and reproductive toxicity (DART) decision tree subcategories from Wu et al. [52], which utilize information on the receptor-binding properties and structural features reported with developmental toxicants to highlight DART substances. We highlighted substances that were reported in the literature as having been tested in the evaluation of alternative test methods for developmental and embryotoxicity [53,54,55,56,57,58,59].

### 2.2. Test Substance Procurement

The screening library of 38 test substances was procured by DTT contractor MRI Global and evaluated for identity and purity. For shipping, the substances were blind-coded and supplied in 1.4 mL polypropylene screw cap vials containing 100 mM stock solutions in DMSO (unless otherwise noted in Supplemental Table S2). Then, 3 of the 38 substances (i.e., aldicarb, bisphenol A, and valproic acid) were randomly chosen to be included twice as test duplicates and serve as an internal control: These additions brought the screening library to 41 blinded test substances. In addition, 3,4-dichloroaniline was provided unblinded to serve as the positive control, creating a 42-substance screening library. Laboratories were also supplied DMSO for further dilutions during testing and to be included on testing plates as a vehicle control, as needed to eliminate variability due to different sources of DMSO. To maintain continuity for the DRF study, all compounds were diluted, prepared for shipping, and stored at −20 °C.

### 2.3. Laboratory Selection

To complete this project, it was determined that a minimum of three laboratories would be selected. Further, those laboratories should represent multiple types of laboratories, including academia, industry, and contract research organizations (CROs), and be capable of performing the technical requirements of the study, including dechorionation of the embryos with acceptable viability (≥20%) per experiment.

Based on those requirements, a request for proposals was developed by DTT and Battelle (a DTT contractor) and subsequently distributed by Battelle to potential zebrafish research labs. The responses to the request for proposals were reviewed and the study laboratories selected. Four laboratories (two academic and two CROs) were selected for the interlaboratory assessment and assigned designations of Lab A, B, C, and D. The data from Lab D are still under evaluation and not included in this publication.

### 2.4. Animal Husbandry

Individual statements are provided for the three laboratories who conducted the studies and provided the data for this publication. Oregon State University: The animal study protocol was approved by Oregon State University’s Institutional Animal Care and Use Committee. ZeClinics: The animal study protocol was approved by the Internal Ethics Committee for Animal Experimentation of the Germans Trias i Pujol Research Institute and by the competent authority. Biobide: The animal study protocol was approved by the Institutional Review Board of Órgano Habilitado of Biodonostia.

### 2.5. Interlaboratory Study Design

The interlaboratory study was conducted in 2 phases: the dose-range-finding (DRF) study and the definitive (Def) study. For the DRF study, the laboratories were allowed to maintain their in-house conditions for testing so that general laboratory performance could be evaluated (i.e., technical issues, time for testing and reporting, etc.) and to help refine the exposure concentrations for the Def study. Importantly, technical challenges can result in increased embryo death and increased variability, which, in turn, can confound the results from chemical exposure. It was our goal to have each of the laboratories perform the DRF assessment with the skills they were most comfortable with prior to conducting a much larger, more complicated Def study.

The laboratories recorded all the study conditions and delivered the individual animal raw data via a template provided by DTT, as well as a summary report of the study findings. The experimental parameters maintained across both phases of the study are described here and the unique aspects of the DRF and Def studies are described below. Each laboratory placed a single zebrafish embryo, at approximately 4–6 h post fertilization (hpf), into individual wells of a 96-well plate for exposure to the test substance, positive control, or vehicle control (0.5% DMSO) in exposure media for 5 days (i.e., 120 hpf). To conduct the exposures, Laboratories A and C used AB strain zebrafish and conducted exposures in E3 media (5 mM NaCl, 0.17 mM KCl, 0.33 mM CaCl2, 0.33 mM MgSO_4_, and 0.1% Methylene Blue) while Laboratory B used the Tropical 5D strain and conducted exposures in E2 media (15 mM NaCl, 0.5 mM KCl, 1.0 mM MgSO_4_, 150 µM KH_2_PO_4_, 50 µM Na_2_HPO_4_, 1.0 mM CaCl_2_, 0.7 mM NaHCO_3_, 0.5 mg/L Methylene Blue). The total volume of the exposure media was 200 µL. All the laboratories used 7 embryos (7 concentrations) for the positive control per plate and 12 embryos for the vehicle control per plate. The substances in the DMSO were dissolved in exposure media to give a final DMSO target concentration of 0.5%. In limited cases, the final DMSO concentration was increased up to a final concentration of 1% to accommodate test substances with low solubility/low stock concentration. Substances were tested at concentrations (7 concentrations at a minimum) up to 100 µM or to their limit of solubility in triplicate. This maximum concentration is comparable to that used by the Padilla laboratory (80 µM) in their ToxCast^TM^ screening [6]. The laboratories were asked to report instances where test substances did not appear to be completely dissolved, as well as the performance of the positive control. The laboratories were also required to have 80% survival of vehicle-control-exposed embryos at 120 hpf on each testing plate; if this condition was not met, the data were discarded, and the test was repeated.

Lastly, all live embryos were visually assessed for mortality at 24 and 120 hpf and for phenotypic alterations at 120 hpf. The laboratories were at liberty to collect data on the phenotypes they saw fit, with most laboratories recording the standard suite of endpoints they typically collected in-house. At a minimum, DTT required that the recordings include edema: presence or absence of swollen pericardial tissue or yolk sacs; craniofacial: presence or absence of defects in the eye, snout, or jaw; axis: curvature of the body axis; trunk: abnormal length; pigment: abnormal, decreased, or absent coloration; and mortality.

### 2.6. Dose-Range-Finding (DRF) Study

**Figure 1:**
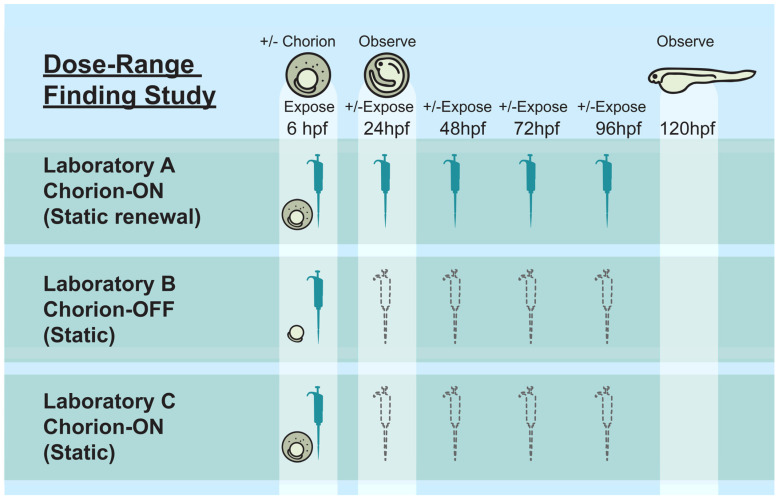
DRF study overview. Schematic representation of the DRF study. Embryos were exposed at 6 h post fertilization (hpf) and, in the case of static renewal, at 24, 48, 72, and 96 h post fertilization (hpf) after the initial exposure. Lab A used chorionated embryos and renewed dosing solutions every 24 h (DRF_Lab A_SR-C). Lab B removed the chorion and used static exposure (DRF_Lab B_S-DC). Lab C used static exposure of chorionated embryos (DRF_Lab C_S-C).

Each laboratory (i.e., Labs A, B, and C) tested a minimum of 7 concentrations between 0.00 and 100.00 µM with individual laboratories determining the dose spacing. For more information regarding the concentrations and dose spacing utilized by each laboratory, please refer to the publication by Hsieh and colleagues (61). Using their in-house exposure protocol for the DRF study, the laboratories exposed embryos under a single exposure condition; static (S) or static renewal (SR) combined with either chorionated (C) or dechorionated (DC) embryos (see Figure 1). Lab A used chorionated embryos and renewed dosing solutions every 24 h (DRF_Lab A_SR-C). For renewal of the exposure media, the embryo media were renewed daily by withdrawing 100 μL of the exposure media and adding 100 μL of 1× working solution, which contained 100 µL E3 media + 100 µL test substance/DMSO solution. This process was repeated 4 times to ensure that the exposure media were properly replenished. Lab B removed the chorion and used static exposure (DRF_Lab B_S-DC). Lab C used static exposure of chorionated embryos (DRF_Lab C_S-DC). Furthermore, 3,4-dichloroaniline was run as a positive control and DMSO as a vehicle control.

The embryo mortality was recorded at 24 and 120 hpf. The incidence of phenotypic alteration(s) representing developmental toxicity was recorded in viable embryos at 120 hpf using the laboratory’s in-house methodology for capturing and evaluating alterations. An incidence of 21, 9, and 12 phenotypic alterations was recorded by Lab A, B, and C, respectively (Table 1).

### 2.7. Definitive (Def) Study Design

**Figure 2:**
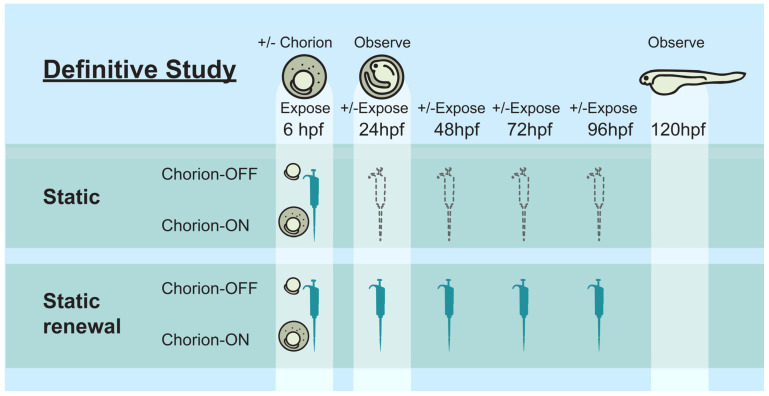
Definitive study overview. Schematic representation of the Def study. The three laboratories participating in the study exposed embryos under four exposure conditions, including static exposure, renewal of exposure media every 24 h, using both chorionated and dechorionated embryos.

The purpose of the Def will be to test the influence of dechorionation and repeated dosing exposure on the developmental toxicity of the test substances. Each laboratory will test a minimum of seven concentrations with the dose selection influenced by the results of the DRF study and feedback from DTT. The laboratories will use the same zebrafish strain, numbers of embryos per test concentration, and exposure media as in the DRF study. As in the DRF study, 3,4-dichloroaniline is the positive control, and DMSO is the vehicle control. The embryo mortality and phenotypic alterations will be recorded, as in the DRF study. Unlike the DRF study, laboratories will be required to expose embryos using the following four exposure conditions in the Def (see Figure 2):

- Static exposure of chorionated embryos (S-C)
- Static renewal exposure of chorionated embryos (SR-C)
- Static exposure of dechorionated embryos (S-DC)
- Static renewal exposure of dechorionated embryos (SR-DC)

This additional Def study will be presented in a future publication.

### 2.8. Data Analysis

The incidence of either dead or malformed embryos at each test substance concentration was converted into a percent response where the denominator was the total number of embryos and the numerator was the number of affected embryos. The incidence of altered phenotypes or dead embryos was used to generate three primary endpoints: *Mortality@24* (i.e., percent of mortality at 24 hpf), *Mortality@120* (i.e., percent of mortality at 120 hpf), and *MalformedAny+Mort@120* (i.e., percent of affected embryos at 120 hpf). An affected embryo was an embryo that was either dead or malformed. The concentration-response data of each plate were analyzed individually using the benchmark concentration (BMC) approach. BMC is comparable to a lowest observed adverse effect level, but is not restricted to the tested concentrations, which facilitates the comparison of results across laboratories using different dose spacing. The BMC approach identifies the point of departure (POD) of the effect using a pre-defined threshold called the benchmark response (BMR). The BMR in this analysis is interpreted as the lowest threshold that provides the best point estimation of the potency based on the intrinsic data variance in an endpoint of a dataset. The BMC/activity call generation is described as follows: For a set of concentration–response data (e.g., incidences of *MalformedAny+Mort@120* at plate#1 treated with ziram at seven different concentrations), 1000 simulated concentration–response curves were generated by bootstrapping the incidences out of total number of animals per concentration and then calculating the percentage of incidence as the response. The simulated concentration–response curves were processed individually using Curvep, a noise-filtering algorithm to detect monotonic response patterns, with an endpoint-specific BMR as the baseline noise parameter. After Curvep processing, the fraction of curves (f) that are not considered purely baseline noise is calculated. If f > 0.5, the effect is considered active, and the BMC plus its confidence interval is calculated using a quantile approach where the BMC value from the noise curves is set as the highest tested concentration [60,61]. The data analysis pipeline is implemented in an R package, Rcurvep (https://cran.r-project.org/package=Rcurvep accessed on 15 January 2024).

Additional statistical analysis was conducted for the data and is presented in the Supplemental Tables. The pairwise Welch’s two-sample *t*-test was conducted using the logarithmic BMC values between pairs of datasets. The analysis was performed to understand whether there was a significant difference between two groups. The Shapiro–Wilk test was conducted to check the normality assumption of the distributions. A non-parametric alternative to the *t*-test, pairwise Wilcoxon test, was conducted when the group sizes were ≥6. The Wilcoxon test was not conducted for smaller group sizes since it is deemed to be insignificant. For inactive substances, the highest tested concentration was used in the analysis. The *p*-value was adjusted using the Bonferroni method. We applied the test based on the BMC data in Supplemental Tables S3b and S8c and the results are reported in Supplemental Tables S3a and S8a,b. Additionally, we conducted a one-way Analysis of Variance (ANOVA) test with Tukey’s post hoc tests using the logarithmic BMC values of the positive control data. The analysis was performed to understand whether there was a significant difference between pairs of groups and to provide an estimate of the difference. The Shapiro–Wilk test was conducted to check the normality assumption of the distributions. A non-parametric alternative to the one-way ANOVA test, a one-way trimmed means test with linear contrast post hoc tests, was conducted. We applied the tests based on the BMC data of the positive control in Supplemental Table S6a and the results are in Supplemental Table S7a,b. The analyses were performed using the rstatix package (https://CRAN.R-project.org/package=rstatix, accessed on 15 January 2024, version 0.7.2) and the *WRS2* package (https://CRAN.R-project.org/package=WRS2 accessed on 15 January 2024) in the R environment.

## 3. Results

The following results summarize the data from the DRF phase of the interlaboratory study to highlight the data collected at the three laboratories using identical test substances but using their in-house protocols, which differed in whether the chorion was removed and whether the exposure media were replenished every 24 h. The data are stored in the SEAZIT relational database created to house the data from both phases of the interlaboratory study [61]. The DRF phase data used in this manuscript have already been made publicly available in Chemical Effects in Biological Systems (CEBS) (https://cebs.niehs.nih.gov/cebs/paper/15646, accessed on 1 May 2023).

### 3.1. Test Substances Characteristics

The test substances are provided in alphabetical order in Table 2. As described in more detail above, all the test substances were selected from the ToxCast^TM^ libraries to represent a broad range of physicochemical and biological properties. The test substances represent uses and structures including drugs, flame retardants, fungicides, herbicides, industrial compounds, insecticides, polycyclic aromatic hydrocarbons, and preservatives [61]. The test substances also represent a wide range of physicochemical properties with octanol–water partition coefficient (logP) ranges from −3.01 to 6.7 and molecular weights ranging from 44.05 to 873.09. We also included a parent compound/metabolite pairing in chlorpyrifos and chlorpyrifos oxon.

The final test substances also have a wide range of biological activities and reference data in support of their selection. Several test substances had previously been tested in zebrafish or in vivo rodent studies, which provides data for future comparisons of the activity of these substances between the current and former studies. Several vascular and endocrine disruptors were selected based on the impact of such substances on development. Test substances were also added based on input from zebrafish researchers that the substance may present some challenges during exposure or may have produced disparate results in their laboratory when the exposure conditions varied. More details on the test substances are as follows:

- A total of 25 substances had test data generated in zebrafish embryos as part of ToxCast^TM^. The activity of the substances ranged from inactive to potent with a median logAC50 value for substances producing phenotypic alterations or mortality of 1.06 µM [62]. In addition, DTT has previously tested 24 of the substances in studies using embryonic zebrafish [48,49,50].
- A total of 26 substances had in vivo developmental toxicity studies conducted in rodents that were available from DTT’s studies, ToxRefDB, or the European Chemicals Agency (ECHA).
- ToxCast^TM^ data were used to assess the potential for test substances to disrupt vascular development [43,44,45]. A total of 31 substances had an evaluation of vascular disruption potential in ToxCast^TM^ testing with a range of activities [45].

A total of three substances are estrogen receptor (ER) pathway agonists and 9 substances are ER antagonists, while 24 substances displayed some degree of ER modulation, indicating activity in at least 1 of 25 assays related to estrogenic activity. A total of seven substances were active for androgen receptor (AR) binding, while 24 substances displayed some degree of AR modulation, indicating activity in at least 1 of 19 assays related to androgenic activity (see Supplemental Table S1). Activity was obtained from the Integrated Chemical Environment v3.7.1 [63].

**Table 2:** Study substances.

Substance Information		Physicochemical Properties ^1^		In Vivo Reference Data			ToxPi Score for Vascular Disruption Markers ^4^	Suggested by Zebrafish Researchers ^5^
Name	CASRN	Molecular Weight	logP	ToxCast^TM^ Zebrafish	Rodent In Vivo Reference Data Identified ^2^	NTP Studies Conducted in Zebrafish ^3^		
3,3′,5,5′-tetrabromobisphenol A	79-94-7	543.87	5.682	Yes	-	Alzualde et al. [48]; Behl et al. [49]	0.27	Yes
3,4-dichloroaniline	95-76-1	162.02	2.37	-	-	-	-	-
6-propyl-2-thiouracil	51-52-5	170.23	0.98	-	Yes	Behl et al. [49]	0.05	-
Abamectin	71751-41-2	873.08	6.61	Yes	-	-	0.33	Yes
Acetaldehyde	75-07-0	44.05	−0.17	-	-	-	-	-
Aldicarb	116-06-3	190.26	1.13	Yes	Yes	Behl et al. [49]; Quevedo et al. [50]	0.07	-
Amoxicillin	26787-78-0	365.40	−3.064	-	-	Behl et al. [49]; Quevedo et al. [50]	-	-
Aspirin	50-78-2	180.16	0.67	-	Yes	Behl et al. [49]	0.03	-
Atrazine	1912-24-9	215.69	2.82	Yes	Yes	-	0.06	-
Bis(tributyltin)oxide	56-35-9	596.11	5.02	-	Yes	Behl et al. [49]; Quevedo et al. [50]	-	-
Bisphenol A	80-05-7	228.29	3.092	Yes	Yes	Behl et al. [49]; Quevedo et al. [50]	0.10	-
Caffeine	58-08-2	194.19	0.16	-	Yes	Behl et al. [49]	0.00	-
Chlorpyrifos	2921-88-2	350.59	0.357	Yes	Yes	Behl et al. [49]; Quevedo et al. [50]	0.10	Yes
Chlorpyrifos oxon	5598-15-2	334.52	3.73	Yes	-	-	0.12	Yes
Dibenz(a,h)anthracene	53-70-3	278.36	6.7	-	-	Behl et al. [49]; Quevedo et al. [50]	-	Yes
Dibutyl phthalate	84-74-2	278.34	NA	Yes	Yes	Behl et al. [49]	0.02	-
Diethylstilbestrol	56-53-1	268.36	5.64	Yes	Yes	Behl et al. [49]; Quevedo et al. [50]	0.30	-
Fluazifop-butyl	69806-50-4	383.37	5.34	Yes	Yes	Behl et al. [49]	0.00	-
Flusilazole	85509-19-9	315.4	4.89	Yes	Yes	-	0.06	-
Hydroxyurea	127-07-1	76.06	−1.606	-	Yes	Behl et al. [49]; Quevedo et al. [50]	0.01	-
Iprodione	36734-19-7	330.17	2.85	Yes	Yes	-	0.06	-
Lindane	58-89-9	290.83	4.26	Yes	Yes	Behl et al. [49]; Quevedo et al. [50]	0.04	-
Linuron	330-55-2	249.1	2.91	Yes	Yes	Behl et al. [49]	0.03	-
Paclobutrazol	76738-62-0	293.79	3.2	Yes	Yes	-	0.03	-
Pentachlorophenol	87-86-5	266.34	4.74	Yes	Yes	-	0.24	-
Phorate	298-02-2	260.37	3.37	Yes	Yes	-	0.03	-
Propofol	2078-54-8	178.27	3.244	-	-	-	-	Yes
Pyrene	129-00-0	202.26	4.93	Yes	-	Quevedo et al. [50]	0.04	Yes
Pyriproxyfen	95737-68-1	321.38	5.55	Yes	Yes	-	0.05	-
Resorcinol	108-46-3	110.11	0.8	-	Yes	-	0.00	Yes
Rotenone	83-79-4	394.42	4.1	Yes	Yes	Behl et al. [49]; Quevedo et al. [50]	0.17	-
Sodium valproate	1069-66-5	166.19	NA	-	-	Quevedo et al. [50]	-	-
Thalidomide	50-35-1	258.24	−0.24	-	-	Behl et al. [49]; Quevedo et al. [50]	0.00	-
Triadimefon	43121-43-3	293.76	2.94	Yes	Yes	Behl et al. [49]	0.03	-
Triclosan	3380-34-5	289.55	4.66	Yes	Yes	-	0.27	-
Triphenyl phosphate	115-86-6	326.29	4.7	Yes	Yes	Alzualde et al. [48]; Behl et al. [49]; Quevedo et al. [50]	0.15	-
Tris(1,3-dichloro-2-propyl)phosphate	13674-87-8	430.91	3.65	Yes	-	Alzualde et al. [48]	-	Yes
Valproic acid	99-66-1	144.22	2.96	Yes	-	Behl et al. [49]	0.00	-
Ziram	137-30-4	305.84	1.29	-	Yes	-	0.30	Yes

^1^ Physicochemical properties were retrieved from ChemSpider. ^2^ Rodent in vivo data were identified in either ToxRefDB, in internal DTT studies, or by the European Chemicals Agency. ^3^ Test substance was previously tested by DTT in zebrafish with results published [48,49,50]. ^4^ ToxPi scores for vascular disruption markers [45]. ^5^ Test substance was recommended by the information group consulted on the conduct of zebrafish embryo screening assays [64]. The source and purity of the study substances is provided in Supplemental Table S2.

### 3.2. DRF Study Results: Summary of Test Substance Activity and Comparison to ToxCast Database

As stated above, the incidence of mortality and altered phenotypes was converted into a percent response, and the response profiles were used to generate a concentration response curve and eventually a BMC. In Table 3, we list the median BMCs of the plates (mostly three, except six for the duplicates) based on the endpoint of *MalformedAny+Mort@120*, which was chosen for presentation because it incorporates both altered phenotypes and mortality. Despite the variations within the laboratory-specific protocols, we can summarize the data trends and point out unique findings within this DRF (Table 3). The potency of substances was compared among substances and across laboratories using a two-sample t-test/Wilcoxon test (when group size ≥ six) plus a normality check of the group distributions, and the results are provided in Supplemental Table S3a. The background data are provided in Supplemental Table S3b.

Out of the 39 test substances, six were inactive at all three laboratories, including 6-propyl-2-thiouracil, acetaldehyde, caffeine, hydroxyurea, resorcinol, and thalidomide. It is interesting to note that all six of the inactive test substances have relatively low logP values and are therefore relatively soluble in aqueous solutions. For the remaining 33 test substances (out of 39) that were active in at least one laboratory, the BMC was the lowest at Lab A for 28 of the 33 test substances; it should be noted that of the 33 substances active in at least one laboratory, 24 were active in all three laboratories. In addition to the substances that were active at multiple laboratories but most potent in Lab A, atrazine, dibenz(a,h)antracene, phorate, and propofol were only active in Lab A (*p*-value < 0.0001 [*t*-test only due to group size = 3] for both Lab A/B and Lab A/C comparisons). Interestingly, the BMC for four substances was lowest at Lab C with two of these substances, aspirin (*p*-value < 0.01 for both Lab A/C and Lab B/C comparisons) and sodium valproate (*p*-value < 0.0001 [*t*-test only due to group size = 3] for both Lab A/C and Lab B/C comparisons), only active in this laboratory. The only substance that was most potent at Lab B was the positive control, 3,4-dichloroaniline (*p*-value < 0.0001 [*t*-test] and *p*-value < 0.01 [Wilcoxon test] to both Lab A/B and Lab B/C comparisons). We also identified five substances (including the positive control) that would need to be tested at lower concentrations in Lab B to generate an accurate BMC since they were active at the lowest test concentration.

We also compared our DRF results with the ToxCast^TM^ zebrafish results available in Supplemental Table S4. When compared with the results for the 25 substances previously tested in ToxCast^TM^, 21 of the substances had the same activity call (active versus inactive) in these three laboratories as what was reported in ToxCast^TM^. It is feasible that some of the discordant test substances could be resolved by testing at higher concentrations; amoxicillin was active in ToxCast and in the current study at Lab A and Lab C, with BMCs above 65 µM, but inactive at Lab B, where it was only tested at up to 64 µM.

### 3.3. Vehicle Control Performance

**Figure 3:**
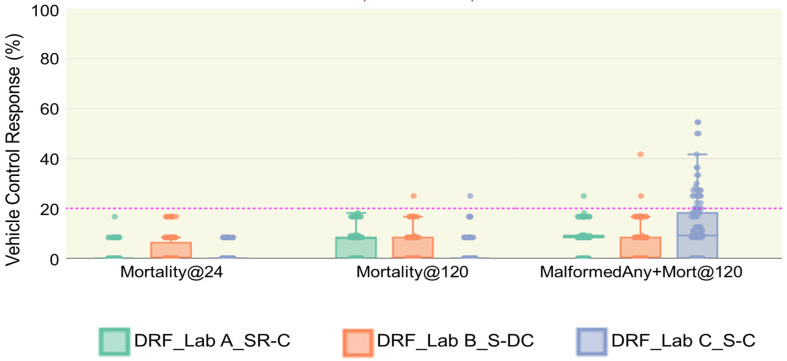
Mortality and altered phenotypes for vehicle-control-exposed embryos. The distribution response (%) of three endpoints (*Mortality@24*, *Mortality@120*, *MalformedAny+Mort@120*) was calculated based on the response (%) from vehicle-control-treated embryos on each plate. The pink horizontal line at 20% represents the upper bound for mortality that is considered acceptable. Each dot represents a plate. The total number of plates per laboratory is 123. The background data are provided in Supplemental Table S5a, and statistics of boxplots are available in Supplemental Table S5b.

A key outcome of the DRF study is that it provides data on test substances and controls that can begin to inform our understanding of reproducibility. The vehicle control (0.5% DMSO) was included on every plate and tested in 12 embryos per plate. One of the criteria for a successful test was a mortality less than 20% in the vehicle-control-exposed embryos at both 24 and 120 hpf. Figure 3 shows the performance of the vehicle control. All plates met the requirement for less than 20% mortality at 24 hpf and only two plates (1 in Lab B and 1 in Lab C) slightly exceeded the 20% mortality threshold in 120 hpf.

Looking more closely at the data, we observed that Lab B had more plates with dead embryos (N = 31) at 24 hpf than Labs A and C (N = 23 and 14, respectively). In addition, 10 of the 31 plates at Lab B had 2 dead embryos while Lab C did not have any plates with more than one dead embryo and Lab A had one plate. Despite the similarity in median values for *Mortality@120* (8.33 µM for Lab A, 0.00 for Lab B and C), the laboratories differed in the number of plates with a dead embryo. At 120 hpf, Lab A had more plates with at least one dead embryo (N = 84) than Lab B (N = 42) and Lab C (N = 29). Within the DRF study, we also observed that the number of vehicle-control-treated embryos with altered phenotypes (i.e., *MalformedAny+Mort@120)* was different across all three laboratories, suggesting that the baseline level of altered phenotypes is higher in Lab C than the other two laboratories. Lab C had 22 plates with three or more affected embryos, while Lab A had only one plate with as many as three affected embryos and Lab B had one plate with three affected embryos and one with five.

### 3.4. Positive Control Performance

**Figure 4:**
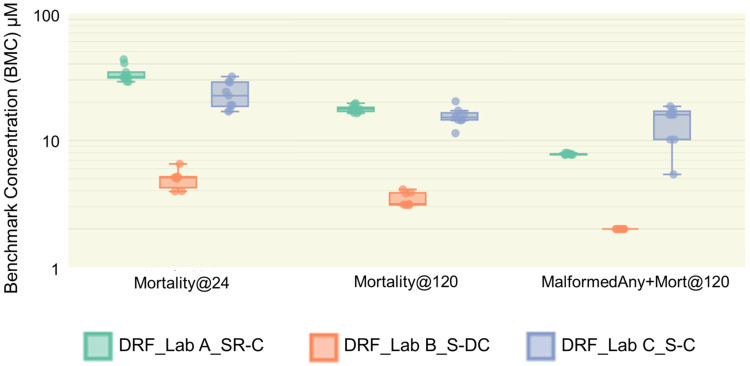
Distribution of BMCs for the positive-control-exposed embryos. The distribution of BMCs for three endpoints (*Mortality@24, Mortality@120, MalformedAny+Mort@120*) was calculated based on the BMC from positive-control-treated embryos on each plate. Each dot represents a BMC derived from the positive control data pooled weekly. N = 10 (Lab A), 7 (Lab B), 9 (Lab C). The background data are provided in Supplemental Table S6a, and statistics of boxplots are available in Supplemental Table S6b.

The positive control, 3,4-dichloroaniline, was run on every plate and the data were pooled weekly to evaluate the assay reproducibility. The data from each week were applied to concentration–response data modeling and a BMC was derived. The BMC distribution is shown in Figure 4 and the median BMC was calculated for each endpoint within each laboratory. The results of the ANOVA test and trimmed means test plus the normality check of group distribution are provided in Supplemental Table S7a,b for between laboratories per endpoint and between endpoints per laboratory comparison, respectively. The BMCs for mortality at 24 hpf were less potent than the later time point of 120 hpf (*Mortality@120* vs. *Mortality@24*: *p*-value < 0.0001 [both tests] for Lab A, *p*-value < 0.001 [for ANOVA test, *p* < 0.01 for trimmed means test] for Lab B, and *p*-value < 0.05 [both tests] for Lab C). At 24 hpf, the median values were 31.79 µM, 5.10 µM, and 22.53 µM in Labs A, B, and C, respectively. At 120 hpf, the median values were 17.68 µM, 3.16 µM, and 15.21 µM in Labs A, B, and C, respectively. On average, the BMC at 120 hpf is 1.88-, 1.43-, and 1.47-fold more potent than at 24 hpf for Lab A, Lab B, and Lab C, respectively. The BMCs calculated for 3,4-dichloroaniline which included mortality and altered phenotypes (i.e., *MalformedAny+Mort@120*) at 120 hpf followed similar patterns as the other endpoints, albeit the BMCs were generally more potent in this combined endpoint (*MalformedAny+Mort@120* vs. *Mortality@120*: *p*-value < 0.0001 [both tests] for Lab A, *p*-value < 0.0001 [for ANOVA test, *p* < 0.01 for trimmed means test] for Lab B, and insignificant [both tests] for Lab C). The median BMC values were 7.82 µM, 2.0 µM, and 15.93 µM in Labs A, B, and C, respectively. Interestingly, Lab C demonstrated greater amounts of variability in the *MalformedAny+Mort@120* endpoint with BMCs ranging from 18.54 µM to 5.40 µM. This high variation in the BMCs across weeks might contribute to the insignificant difference in BMCs between the *MalformedAny+Mort@120* endpoint and the *Mortality@120* endpoint in Lab C. The positive control was once again most potent at Lab B with a BMC of 2.0 µM using the *MalformedAny+Mort@120* endpoint and the lack of variability at that laboratory was due to its high potency (all embryos were affected at the lowest test concentration), resulting in a need for retesting at lower test concentrations at that laboratory in order to assess potency. On average, the BMCs of Lab B in the *MalformedAny+Mort@120* endpoint were 3.89- and 6.60-fold more potent than Lab A and Lab C, respectively (*p*-value < 0.0001 [both tests]).

### 3.5. Reproducibility of Duplicate Test Substances

Aldicarb, bisphenol A, and valproic acid were randomly selected for an assessment of reproducibility within a given laboratory’s testing protocol. Each laboratory screened the same substance twice (i.e., duplicates), and each time, three plates were screened. In total, six BMC values can be derived for each of the three substances. Based on these data, we can investigate the intralaboratory reproducibility and interlaboratory reproducibility. Table 4 lists the BMC values of the *Mortality@120* and *MalformedAny+Mort@120* endpoints of the three substances. The results of the t-test test and normality check of group distributions are provided in Supplemental Table S8a,b. Supplemental Table S8a reports the *t*-test results for a comparison between the duplicated test substances per endpoint–laboratory and Supplemental Table S8b reports the *t*-test results for comparisons between laboratories per endpoint-duplicated test substance. The background data for the *t*-tests are available in Supplemental Table S8c. Since no group size was greater than six, the result of the non-parametric Wilcoxon test is deemed to be insignificant, so the Wilcoxon test was not conducted.

The BMCs for the *Mortality@120* endpoint alone were generally consistent between plates and between duplicates within a given laboratory; if there was mortality with one duplicate, there was mortality with the second, and the BMCs were comparable. Two exceptions were observed: one is aldicarb from Lab A and the other one is valproic acid from Lab C. However, for the aldicarb from Lab A, the discordance might be related to the fact that the effect occurred close to the highest tested concentration. Lab C was the only laboratory that recorded mortality for valproic acid, and the BMC varies both between plates and between duplicates. For example, the BMC values from duplicate#1 of valproic acid varied from 3.63 to 11.47 µM between plates, and for duplicate#2, the BMC values varied from 6.54 to 38.18 µM between plates. However, the BMC difference in the two listed exceptions is not significant.

The addition of phenotypic alterations (i.e., the *MalformedAny+Mort@120* endpoint) produced more potent BMCs. The BMCs of aldicarb and bisphenol A were consistent within each of the three laboratories. Valproic acid showed less consistency within each of the three laboratories. At Lab A, duplicate#1 was active in all three plates with BMCs ranging from 46.65 to 58.17 µM, while duplicate#2 was inactive in all plates. At Lab B, valproic acid duplicate#1 had a single plate with activity and a BMC of 87.87 µM, while duplicate#2 was inactive. In contrast, at Lab C, valproic acid in all six plates were active and with potent BMCs ranging from 0.98 to 20.94 µM. The BMC difference between duplicated test substances is significant for aldicarb in Lab B (*p*-value < 0.05) and for valproic acid in Lab A (*p*-value < 0.01).

These data also provide insight into the interlaboratory variability, which may reflect differences in the protocols used by the three laboratories. For the *Mortality@120* endpoint, bisphenol A was consistently active in all plates, all duplicates, and all three laboratories with comparable BMC values. Aldicarb was generally inactive while valproic acid was only active in Lab C despite there being varying BMC values between plates. The BMC difference between Lab A/B and Lab C is significant in both duplicated test substances of valproic acid (*p*-value < 0.001 for duplicate#1 and *p*-value < 0.05 for duplicate#2). Using the *MalformedAny+Mort@120* endpoint, for aldicarb and bisphenol A, the BMC values of Lab A are significantly lower than Lab B in all duplicates except duplicate#1 of aldicarb. The BMC difference between Lab A and Lab C for aldicarb and bisphenol A is insignificant except duplicate#2 of aldicarb (*p*-value < 0.01). For valproic acid, the interlaboratory results varied in the *MalformedAny+Mort@120* endpoint. Valproic acid was potently active in Lab C, inactive in Lab B, and inconclusive in Lab A. The BMC difference is significant between Lab A/B and Lab C of both duplicates of valproic acid.

### 3.6. Test Substance Interlaboratory Variability

In the DRF study, laboratories used their in-house exposure protocols, which varied according to whether the chorion was removed, as well as in whether the exposure media were renewed every 24 h. As such, we anticipated that an across-laboratory comparison of the substance potency would provide discordant or inconsistent results. To evaluate this hypothesis, we utilized data collected from the *MalformedAny+Mort@120* endpoint to compare the potency ranking of substances across laboratories.

**Figure 5:**
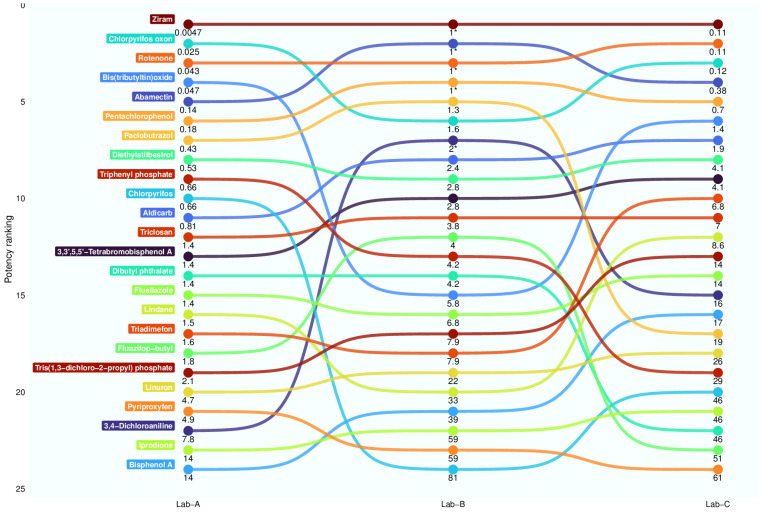
Comparison of potency for 24 test substances active at all laboratories. The bump chart is based on potency ranking of substances that produced phenotypic alterations or mortality (the *MalformedAny+Mort@120* endpoint) at each study laboratory. The value presented below each circle represents the median BMC for that test substance within a laboratory. A line was drawn connecting the median BMCs for each test substance within the three laboratories. Each test substance was randomly given a different color to assist with differentiating between test substances. An “*” next to a BMC value indicates that the BMC reflects the lowest test concentration, and the substance would need to be retested at lower concentrations to generate a more accurate BMC.

For each substance, the median was used to summarize the BMC values of multiple plates (Table 3). Only substances that were active in all three laboratories were used in the following analysis. For each laboratory, the ranks were generated based on the BMC values of the 24 substances. Then, the ranking lists from three laboratories were visualized (Figure 5), and the ranking statistics are available in the Supplemental Table S9.

As seen in Figure 5, 24 substances produced mortality and/or phenotypic alterations in all three laboratories. Based on the average ranks across three laboratories, ziram (mean = 1.33), rotenone (mean = 2.67), chlorpyrifos oxon (mean = 3.67), abamectin (mean = 3.67), and pentachlorophenol (mean = 5.00) were the top five most consistently potent substances regardless of laboratory, while pyriproxyfen (mean = 22.67), iprodione (mean = 22.00), and bisphenol A (mean = 20.33) were the top three substances with the lowest potencies across laboratories.

The substances with the top five least variability in potency ranks across laboratories, in terms of standard deviation (SD) of the ranks, were ziram (SD = 0), rotenone (SD = 0.58), diethylstilbestrol (SD = 0.58), triclosan (SD = 0.58), and pentachlorophenol (SD = 1.00). Ziram, rotenone, and pentachlorophenol had higher ranks than diethylstilbestrol and triclosan.

The top five most variable substances across laboratories in terms of potency ranks were 3,4-dichloroaniline (SD = 7.51), chlorpyrifos (SD = 7.21), paclobutrazol (SD = 6.43), bis(tributyltin)oxide (SD = 5.86), and fluazifop-butyl (SD = 5.51). 3,4-dichloroaniline, chlorpyrifos, and fluazifop-butyl had varying ranks between laboratories but bis(tributyltin)oxide and paclobutrazol had a similar ranking among two laboratories. For example, paclobutrazol ranked 7th, 5th, and 17th for Lab A, Lab B, and Lab C, respectively.

Overall, the data generated and described in each section of the results help demonstrate a realistic range of variability that may be observed within a laboratory setting (i.e., intralab) and across labs (i.e., interlab) when in-house protocols are utilized.

## 4. Discussion

Zebrafish embryos have become a popular model to screen chemicals for various toxicological endpoints. Around the time Brannen et al. [22] coined the term “zebrafish embryo teratogenicity test” (ZET), this marked a period of rapid increase in using zebrafish embryos to screen chemicals for developmental toxicity. A few years later, Beekhuijzen et al. [24] reviewed the use of zebrafish embryos in screening by compiling their in-house experience with a survey of the literature and concluded that, despite the multitude of scoring systems that had been developed, the activity calls (active or inactive) were generally consistent across laboratories, while the reported potencies (POD, BMC, LC_50_, etc.) of the test substances varied because of experimental design differences.

With the lessons learned from our previous efforts to understand the variability in the toxicological outcomes associated with the embryonic zebrafish model [64], we designed the current study in two phases: the DRF and Def. We conducted the DRF study to establish the appropriate working concentrations of the test substances to prepare for our Def study, in which we plan to evaluate the role of the chorion and exposure frequency in test substance potency. Although not the primary goal of this interlaboratory study, comparing our results to other databases (in vitro, zebrafish, *C. elegans*, or rodents) will allow us to better understand the performance of zebrafish as a model species for developmental or general toxicity relative to other model species. We can look at concordance by activity call or by potency, or if we have enough data, we can compare the phenotypic data across other zebrafish or rodent studies. As a starting point, the preliminary data from the DRF were compared to the available literature on zebrafish studies or rodent developmental toxicology studies to assess the concordance. We learned that all three laboratories had the same activity call for 21 of 25 substances also tested in ToxCast^TM^ (Supplemental Table S4). We will expand these types of assessments following the collection of the Def study results since it is a more robust dataset to work with.

Here, we discuss some of our preliminary lessons learned from the DRF, as well as our expectations and hypotheses for the Def interlaboratory study results, which will be provided in future publications. In the current DRF study, the laboratories could use many of their in-house methods to avoid the technical challenges of adopting a new protocol. In the current study, 1 laboratory conducted exposure utilizing chorion-on with static renewal, while the other two laboratories used chorion-on or chorion-off combined with static exposure. While this strategy might seem like a limitation, it allowed us to identify the commonalities and differences in the laboratories’ screening protocols, as well as understand the variety of approaches to data analysis and interpretation that we received as laboratory reports. The Def study will allow us to better understand the influence of the chorion and exposure frequency as well as determine other interlaboratory differences that could affect test substance potency. In our previous exercise, a group of zebrafish researchers suggested studying the influence of the chorion status and frequency of exposure on the test substance activity, although several other factors, including zebrafish strain, exposure apparatus, method of chemical preparation and delivery, phenotypes scored, and scoring system, were discussed at length as factors that may also influence the test substance activity [64]. Even though we are focusing the Def study on two experimental variables, we have collected enough information that the multiple exposures conducted in each of the laboratories will allow us to hypothesize whether other experimental parameters could play a role in toxicity responses [65]. For example, rodent studies have shown that the strain of rodent used influences the outcome of toxicity studies, and it is worth understanding whether this is the case using zebrafish as a model species [66,67,68,69]. While more work is required to understand the differences among zebrafish strains, limited reports have demonstrated strain differences following exposure to ethanol [70,71]. In the current study, two of the laboratories use the AB strain while the other laboratory uses Tropical 5D, in addition to differences in the exposure conditions. Besides strain differences, Truong et al. [25] demonstrated that the chemical delivery methods can greatly influence the water concentration and toxicity of a chemical. The authors demonstrated that digital dispensing produced greater reproducibility than traditional pipet delivery of test chemicals and assert that the utilization of more consistent delivery methods should increase the reproducibility across laboratories. In the current study, one laboratory used digital dispensing for chemical exposure, while the other laboratories used more traditional pipet delivery. These differences should allow us to gain insights into factors beyond the chorion and dosing frequency for future studies.

In the DRF, despite differences in whether the chorion was present or removed, exposure frequency, as well as differences in the exposure media, zebrafish strain, and other exposure parameters, there was reasonable agreement across the three laboratories in terms of the activity of the test substances. Of the 39 test substances, 30 had the same activity call, while 33 of 42 (78.6%) test substances did when duplicate chemicals were included (Table 3: active versus inactive for *MalformedAny+Mort@120*) in all three laboratories. Karmaus et al. [72] examined the data variability in a regulatory required in vivo test, the rat oral LD50 test, and reported that replicate studies only produced the same hazard categorization approximately 60% of the time because of biological or protocol variability. Given the differences in exposure parameters, this variability is consistent with reports from select rodent in vivo studies and is consistent with Beekhuijzen et al. [24].

Beyond active versus inactive outcomes across laboratories, comparing the potency outcomes from the DRF allows us to better categorize the variability within this model. To begin, we evaluated the performance of the vehicle and positive control. We hypothesized that substantial mortality would not occur in embryos exposed to the vehicle control (0.5% DMSO) within and across laboratories, but that it was possible for vehicle control fish to have varied background altered phenotypes. As expected, we observed that all three laboratories generally had mortality rates below the required 20% incidence rate, which initially suggested that there were no issues with the experimental setups in each laboratory. However, a closer look at the results highlights that Lab B had more plates with two dead embryos, 10 plates versus none and a single plate at the other two laboratories (Supplemental Table S5a). These slight differences in mortality rates across laboratories may be because of a variety of husbandry or experimental reasons which are currently unknown, but overall, this result does not seem to correlate with the incidence of altered phenotypes seen in the *MalformedAny+Mort@120* endpoint of the vehicle control embryos. The incidence of malformations at 120 hpf in Lab C suggests potential issues with animal husbandry or the recording of altered phenotypes, as this laboratory had 30 plates with greater than 20% affected embryos while the other two laboratories had at most two plates with this high an incidence of affected embryos. The variability seen in *MalformedAny+Mort@120* at Lab C will be evaluated further in the definitive study.

Besides the variability in the background incidence of phenotypic alterations, the response to the positive control showed interlaboratory variability (Supplemental Table S7a). This was particularly evident in the *Mortality@120* endpoint, where Lab B consistently reported that 3,4-dichloroaniline was far more potent at producing mortality, with a median BMC of 3.16 µM, compared to BMCs of 17.68 µM and 15.21 µM at Lab A and Lab C, respectively. This potency at Lab B also resulted in it being ranked more potent relative to other test articles; 3,4-dichloroaniline ranked seventh at Lab B versus 22nd and 15th at the other laboratories (Figure 5; Supplemental Table S9). Schiwy et al. [73] conducted an uptake study of 3,4-dichloroaniline using static renewal of the exposure media in embryonic zebrafish and reported that the chemical rapidly dissipates over a 24 h period. Since Lab A used static renewal, one might expect the replenishment of solution would produce greater potency for 3,4-dichloroaniline, which was not the case. It is possible that the first 24 h of exposure represents a critical period of development for exposure to 3,4-dichloroaniline, although it is also possible other differences in methodology account for these inconsistencies. Interestingly, Lab B was the only laboratory to remove the chorion in the DRF study, so perhaps that affected the potency. We anticipate that the Def study will provide a wealth of data on negative and positive controls for future intralaboratory and interlaboratory comparisons and it will be important to try to understand the variability in the positive control, as that variability complicates the interpretation of data.

We also included three test substances (bisphenol A, aldicarb, and valproic acid) as blinded duplicates to further assess the variability (Table 4, Supplemental Table S8a,b). Bisphenol A produced similar BMCs for mortality and phenotypic alterations across the three laboratories. Aldicarb produced phenotypic alterations at all three laboratories and at similar concentrations; however, aldicarb only produced mortality at Lab A at high exposure concentrations. Interestingly, since the laboratories used their in-house protocols in the DRF study, Lab A was the only laboratory that renewed the exposure solution every 24 h, and perhaps this is what resulted in greater toxicity. Lab A also reported the most potent BMC values for the 28 of 33 test substances that were active in at least 1 laboratory. Interestingly, valproic acid produced mortality in all replicates tested at Lab C but did not produce any mortality at the other laboratories. Since Lab C ran static exposure of chorionated embryos, it is unclear why valproic acid was more toxic in this laboratory. It is anticipated that the Def phase of the study should provide valuable insights into the influence of the exposure design on the potency of these blinded duplicates.

Beyond the variations in the exposure parameters, the laboratories vary substantially in the number of endpoints measured, which endpoints are measured, how the endpoints are defined, and the labels applied to those endpoints [24,39,65]. The discordant phenotype screening lists among Labs A, B, and C (Table 1) also can have an impact on the outcomes for a more detailed analysis. One of the first issues we encountered in attempting to compare the results from the DRF by phenotype is that each laboratory has their own terminology; the phenotypes varied in number, in the label applied to them, and the definition. Among the phenotypes requested was “Craniofacial: presence or absence of defects in eye, snout, or jaw”. In response to that request, Lab A measured five phenotypes in the craniofacial region, although none of those used the word craniofacial as a label. Lab B reported data as a single composite phenotype labeled “Defects in the Craniofacial region”, whereas Lab C reported 5 phenotypes with “craniofacial” as a portion of the label. To create consistent terminology in the current study, the phenotypes were discussed with the study laboratories and mapped to standard terminology using the Ontology Search website (https://www.ebi.ac.uk/ols/index accessed on 15 January 2024) [61]. This also led us to investigate the use of ontologies to help us harmonize the lab-specific terms. In our recent collaboration, Thessen et al. [74] conducted a two-part exercise in which zebrafish researchers assessed images using in-house terminology for altered phenotypes, followed by the same assessment using the standardized terminology provided to them. The authors concluded that use of standardized terminology inherently improves heterogeneity and increases the agreement and repeatability between laboratories. The variability in the phenotype data among laboratories may make the interpretation of data challenging. Greater uniformity in phenotype definition and terminology should foster broader acceptance of this model.

Consistent results from a method are critical to the acceptance and utility of a model and the data generated [38]. As more screening data are generated using the zebrafish embryo model, it is apparent that inconsistencies between laboratories in activity calls or measures of potency of chemicals exist. For example, Wilson et al. [26] screened a small set of chemicals using four exposure regimens and reported shifts in the potency of chemicals based on how the exposure was conducted. These results led the authors to conclude that much of the difference in activity call or potency is due to protocol differences, and that a standardized exposure regimen is not only achievable but would promote the utility of data. Similarly, Hsieh et al. [61] compared data generated using different protocols and found that the concordance dropped when comparing data prepared using different protocols. The potential contributing protocol parameters that shifted the potency included fish strain, chorionation status, static exposure scenario, exposure volume, and the time-point at which endpoints are measured. As in Wilson et al. [26], Hsieh et al. [61] concluded that much of this inconsistency in test results between laboratories was due to differences in the methodology used to test the chemicals. The findings in the DRF study (which mimics a real-world comparison of study results across laboratories) reiterate and support the need for a more thorough evaluation of the impact of experimental parameters on the study outcome. Conclusions and recommendations for protocol harmonization and discussion regarding the impact of these results on the toxicology community are not advised based upon the study design. These valuable discussion points, which are the goals of the interlaboratory study, will be generated following review of the Def study results.

To build upon our work and that of others, we designed the Def phase of this study to further elucidate the role of the chorion and exposure media renewal on the activity calls and potency. Future publications from the Def study will (1) confirm and quantify whether these protocol parameters have a notable impact on the chemical potency; (2) provide a more robust assessment of the variability within and across laboratories, which will help establish a baseline for improvement with protocol harmonization; (3) provide insight into chemical-specific phenotypes that could direct future mechanistic research; and (4) utilize standardized ontology terms to showcase their value for comparing developmental toxicity phenotypes across zebrafish laboratories, as well as other species. Since the laboratories taking part in the Def study will use different means of chemical preparation and delivery, zebrafish strains, phenotypes scored, and scoring systems, the interlaboratory comparison should also provide insights into additional components of the experimental design to control for more consistent results. Ultimately, we aim for this work to provide a foundation for critical discussions surrounding recommendations for protocol harmonization to increase confidence in this model and facilitate its broader adoption by the toxicology community.

## Figures and Tables

**Table 1:** Phenotypes recorded by the laboratories.

Lab A	Lab B	Lab C
Abnormal_heartbeat	Abnormal axial bend (AXIS)	Axis__curvature_of_body_axis
Abnormal_length	Abnormal brain region (BRN_)	Craniofacial__edema
Abnormal_pigmentation	Abnormal notochord (NC__)	Craniofacial__jaw_defects
Absence_heartbeat	Abnormal swim bladder, muscle pattern, blood circulation (MUSC)	Craniofacial__snout_defects
Altered_jaw_morphology	Abnormal touch response in the caudal fin (TCHR)	fin_absence
Altered_snout	Defects in the craniofacial region (CRAN)	necrosis
Curved_axis	Defects in the lower trunk region (LTRK)	notochord_defect
Decreased_absent_pigmentation	Defects on the skin (SKIN)	otoliths_defects
Delayed_Hatching	Edema of the heart, yolk sac or brain region (EDEM)	scoliosis
Malformed__disorganized_or_missing_somites		tail_bending
Malformed_or_missing_caudal_fin		Unhatched
Malformed_or_missing_otic_vesicle		Yolk_sac__Edema
Malformed_or_missing_trunk		
Notochord_malformation		
Others		
Presence_of_head_Edema		
Presence_of_pericardial_Edema		
Smaller_abnormal_eye_shape		
Smaller_abnormal_head_shape		
Yolk_opacity		
Yolk_sac_Edema		

Note: Phenotypes that catalog abnormal development are written as provided by the laboratories. Note: Only phenotypes that were used in the calculation of *MalformedAny+Mort@120* are listed. For more information regarding the phenotypes utilized by each laboratory and how they align, please refer to the publication by Hsieh and colleagues (61).

**Table 3:** Median BMCs for *MalformedAny+Mort@120* endpoint in DRF data.

Substance	CASRN	Lab A_SR-C	Lab B_S-DC	Lab C_S-C
3,3′,5,5′-tetrabromobisphenol A	79-94-7	1.40 ^1^	2.80	4.10
3,4-dichloroaniline	95-76-1	7.80	2.00 *	16.00
6-propyl-2-thiouracil	51-52-5	Inactive (100)	Inactive (100)	Inactive (100)
Abamectin	71751-41-2	0.14	1.00 *	0.38
Acetaldehyde	75-07-0	Inactive (100)	Inactive (100)	Inactive (100)
Aldicarb ^2^	116-06-3	0.81	2.40	1.90
Amoxicillin	26787-78-0	81.00	Inactive (64)	65.00
Aspirin	50-78-2	Inactive (100)	Inactive (100)	14.00
Atrazine	1912-24-9	49.00	Inactive (100)	Inactive (100)
Bis(tributyltin)oxide	56-35-9	0.047	5.80	1.40
Bisphenol A ^2^	80-05-7	14.00	39.00	17.00
Caffeine	58-08-2	Inactive (100)	Inactive (100)	Inactive (100)
Chlorpyrifos	2921-88-2	0.66	81.00	46.00
Chlorpyrifos oxon	5598-15-2	0.025	1.60	0.12
Dibenz(a,h)anthracene	53-70-3	0.081	Inactive (64)	Inactive (100)
Dibutyl phthalate	84-74-2	1.40	4.20	46.00
Diethylstilbestrol	56-53-1	0.53	2.80	4.10
Fluazifop-butyl	69806-50-4	1.80	4.00	51.00
Flusilazole	85509-19-9	1.40	6.80	14.00
Hydroxyurea	127-07-1	Inactive (100)	Inactive (100)	Inactive (100)
Iprodione	36734-19-7	14	59	46
Lindane	58-89-9	1.5	33	8.6
Linuron	330-55-2	4.7	22	26
Paclobutrazol	76738-62-0	0.43	1.3	19
Pentachlorophenol	87-86-5	0.18	1 *	0.7
Phorate	298-02-2	1.7	Inactive (100)	Inactive (100)
Propofol	2078-54-8	0.49	Inactive (100)	Inactive (100)
Pyrene	129-00-0	4.3	39	Inactive (100)
Pyriproxyfen	95737-68-1	4.9	59	61
Resorcinol	108-46-3	Inactive (100)	Inactive (100)	Inactive (100)
Rotenone	83-79-4	0.043	1 *	0.11
Sodium valproate	1069-66-5	Inactive (100)	Inactive (100)	4.1
Thalidomide	50-35-1	Inactive (100)	Inactive (100)	Inactive (100)
Triadimefon	43121-43-3	1.6	7.9	6.8
Triclosan	3380-34-5	1.4	3.8	7
Triphenyl phosphate	115-86-6	0.66	4.2	29
Tris(1,3-dichloro-2-propyl) phosphate	13674-87-8	2.1	7.9	14
Valproic acid ^2^	99-66-1	76	Inactive (100)	4.1
Ziram	137-30-4	0.0047	1 *	0.11

^1^ BMC values for *MalformedAny+Mort@120* endpoint expressed in µM. ^2^ Test substance run in duplicate. * Indicates that the substance would need to be retested at lower concentrations to obtain a BMC. For a given test substance, the grey shaded cell has the lowest BMC among the three laboratories.

**Table 4:** BMC values of *MalformedAny+Mort@120* and *Mortality@120* endpoints for duplicated test substances.

	*Mortality@120*			*MalformedAny+Mort@120*		
	Lab-A_SR-C	Lab-B_S-DC	Lab-C_S-C	Lab-A_SR-C	Lab-B_S-DC	Lab-C_S-C
Aldicarb Duplicate #1	Inactive ** 90.45 90.45	Inactive Inactive Inactive	Inactive Inactive Inactive	1.39 * 0.53 1.44	1.32 1.42 2.24	2.09 1.31 2.58
Aldicarb Duplicate #2	90.45 86.41 Inactive	Inactive Inactive Inactive	Inactive Inactive Inactive	0.81 0.81 0.58	2.52 2.52 3.58	2.32 1.57 1.75
Bisphenol A Duplicate #1	40.54 40.54 40.54	61.05 79.16 55.75	38.18 38.18 38.18	13.90 14.37 13.90	32.86 40.29 39.49	45.73 18.19 19.24
Bisphenol A Duplicate #2	40.54 *** 40.54 40.54	55.75 58.52 46.38	38.18 38.18 17.47	13.90 13.90 8.10	39.49 39.49 39.49	16.45 14.48 16.45
Valproic Acid Duplicate#1	Inactive Inactive Inactive	Inactive Inactive Inactive	11.47 11.47 3.63	46.65 58.17 58.17	Inactive Inactive 87.87	0.98 4.12 4.12
Valproic Acid Duplicate #2	Inactive Inactive Inactive	Inactive Inactive Inactive	11.47 38.18 6.54	Inactive Inactive Inactive	Inactive Inactive Inactive	20.94 1.75 4.12

* For each cell in the table, BMC values (µM) came from three plates. ** The highest tested concentration for all inactive substances is 100 µM. *** Identical BMCs are due to identical response data near BMR.

## Data Availability

The data are available in Chemical Effects in Biological Systems (CEBS) and can be downloaded through this link: https://doi.org/10.22427/NTP-DATA-002-00102-0001-000-6 (accessed on 15 January 2024).

## Supplementary Materials

The following supporting information can be downloaded at: https://www.mdpi.com/article/10.3390/toxics12010093/s1, Within this excel file, each tab contains separate information including Table S1: Test substance endocrine disruption data obtained from the literature and ICE (Integrated Chemical Environment) https://ntp.niehs.nih.gov/go/niceatm-ice accessed on 15 January 2024; Table S2: Test substance list with procurement information and identifiers; Table S3a: Comparing potency of substances across labs: Pairwise statistics tests results for BMC values of *MalformedAny+Mort@120* endpoint; Table S3b: Background data for statistical tests in Table S3a; Table S4: Comparison of test substance activity to testing in ToxCastTM and data used to make the calculations; Table S5a: Incidence of mortality and phenotypic alterations in vehicle control treated embryos; Table S5b: Incidence of mortality and phenotypic alterations in vehicle control treated embryos-median and quartiles; Table S6a: BMCs for mortality and phenotypic alterations in positive control treated embryos (background data for statistical tests in Table S7a,b); Table S6b: BMCs for mortality and phenotypic alterations in positive control treated embryos-median and quartiles; Table S7a: Pairwise statistical tests results for positive control (between laboratories per endpoint); Table S7b: Pairwise statistical tests results for positive control (between endpoints per laboratory); Table S8a: Pairwise statistical tests results for BMC values for duplicated test substances (between duplicated test substances per endpoint-laboratory); Table S8b: Pairwise statistical tests results for BMC values for duplicated test substances (between laboratories per endpoint-duplicated test substance); Table S8c: Background data for statistical tests in Table S8a,b; Table S9: Test substance activity rankings based on median BMC values for 24 test substances active in all three laboratories.
